# Comprehensive Profiling of Roquin Binding Preferences for RNA Stem‐Loops

**DOI:** 10.1002/anie.202412596

**Published:** 2024-11-06

**Authors:** Lasse Oberstrass, Jan‐Niklas Tants, Chiara Lichtenthaeler, Sara E. Ali, Louisa Koch, David H. Mathews, Andreas Schlundt, Julia E. Weigand

**Affiliations:** ^1^ Department of Pharmacy, Institute of Pharmaceutical Chemistry University of Marburg Marbacher Weg 6 35037 Marburg Germany; ^2^ Institute for Molecular Biosciences and Biomolecular Resonance Center (BMRZ) Goethe-University Frankfurt Max-von-Laue-Str. 7–9 60438 Frankfurt Germany; ^3^ Department of Biochemistry & Biophysics and Center for RNA Biology University of Rochester Medical Center Rochester NY 14642 USA; ^4^ Institute of Biochemistry University of Greifswald Felix-Hausdorff-Str. 4 17489 Greifswald Germany

**Keywords:** RNA structures, RNA-binding proteins, RNA interactome, RNA–protein interactions

## Abstract

The cellular levels of mRNAs are controlled post‐transcriptionally by *cis*‐regulatory elements located in the 3’‐untranslated region. These linear or structured elements are recognized by RNA‐binding proteins (RBPs) to modulate mRNA stability. The Roquin‐1 and −2 proteins specifically recognize RNA stem‐loop motifs, the trinucleotide loop‐containing constitutive decay elements (CDEs) and the hexanucleotide loop‐containing alternative decay elements (ADEs), with their unique ROQ domain to initiate mRNA degradation. However, the RNA‐binding capacity of Roquin towards different classes of stem‐loops has not been rigorously characterized, leaving its exact binding preferences unclear. Here, we map the RNA‐binding preference of the ROQ domain at nucleotide resolution introducing sRBNS (structured RNA Bind‐n‐Seq), a customized RBNS workflow with pre‐structured RNA libraries. We found a clear preference of Roquin towards specific loop sizes and extended the consensus motifs for CDEs and ADEs. The newly identified motifs are recognized with nanomolar affinity through the canonical RNA‐ROQ interface. Using these new stem‐loop variants as blueprints, we predicted novel Roquin target mRNAs and verified the expanded target space in cells. The study demonstrates the power of high‐throughput assays including RNA structure formation for the systematic investigation of (structural) RNA‐binding preferences to comprehensively identify mRNA targets and elucidate the biological function of RBPs.

## Graphical abstract

sRBNS (structured RNA Bind‐n‐Seq) allows precise mapping of RNA‐protein interactions including RNA structures at nucleotide resolution. Using this new method, we dissected the RNA‐binding preferences of Roquin proteins towards RNA stem‐loops, identified new high‐affinity motifs and expanded the mRNA target space in cells. Thus, sRBNS allows for the detailed investigation of complex binding events beyond single stranded RNA motifs.

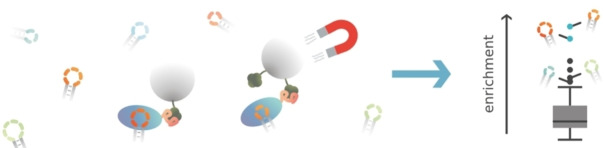

## Introduction

The human genome encodes an unexpectedly large number of RNA‐binding proteins (RBPs). Based on the eukaryotic RBP database (EuRBPDB) approximately 3,000 RBPs are present in human cells.^[1]^ However, only a minor fraction of these RBPs have been systematically studied, leaving it unclear which RNAs they bind to and what their biological function is. Where RNA‐binding preferences are known, they are often short, linear sequence motifs. Yet, RNA has the ability to form intricate structures that are essential for its cellular function and can be specifically recognized by RBPs.^[2]^ However, identifying functional structures in the transcriptome is more challenging than finding sequence matches, so there are only few detailed examples of how RBPs interact with a specific RNA structure.^[3]^

In mRNAs, most functional structural elements are located in the untranslated regions (UTRs). Here they act as *cis*‐regulatory elements and play a crucial role in post‐transcriptional gene regulation, affecting pre‐mRNA splicing, cellular localization, translation rate, and mRNA stability.^[4]^ Therefore, they allow for the precise adaptation of the proteome to changing internal and external signals. To allow for individual levels of target control, mRNAs contain distinct arrangements of high and low affinity motifs within a specific context, e.g. in close proximity to binding sites of other RBPs to allow for combinatorial control. As a result, subtle sequence and/or structural differences in binding motifs determine whether and how efficiently a target mRNA is recognized and thus regulated by a specific RBP. High‐throughput methods are necessary to comprehensively catalogue possible binding motifs for a given RBP in order to define its target hierarchies and thus, its impact on cellular pathways.

**Figure 1:**
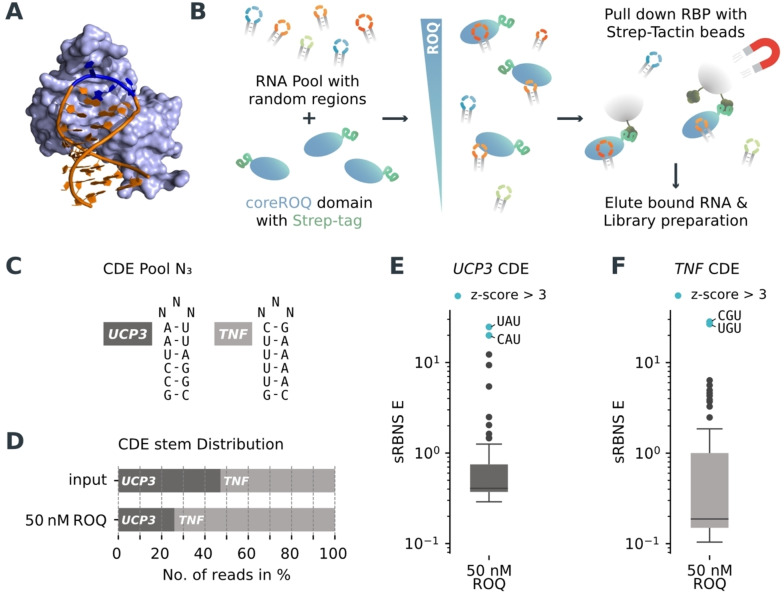
sRBNS recapitulates known CDE preferences. A) Example structure of the protein‐RNA complex of the Roquin ROQ domain with the CDE 1 stem‐loop of *UCP3* (PDB: 6TQB^**[20]**^). B) Principle of an sRBNS experiment for high‐throughput identification of protein interactions with RNA structures. C) Experimental design with two CDE stem‐loops of the 3’‐UTR of *UCP3* and *TNF* with three random loop nucleotides as input library and 50 nM ROQ domain of Roquin‐1. D) Distribution of the different stems in the input library and the pulldown library. E and F) Boxplot of the enriched motifs in the trinucleotide loops of *UCP3* (E) and *TNF* (F) stems, respectively. Shown is the enrichment (sRBNS E) against the input at 50 nM ROQ domain concentration. Pool size *n*=64. Values greater than three standard deviations above the mean are considered significantly enriched and highlighted in blue (z‐score >3).

Roquin proteins are model RBPs for studying protein interactions with RNA structures. The Roquin‐1 and −2 proteins (encoded by *RC3H1 and RC3H2*) harbor a unique ROQ domain that allows them to recognize the shape of stem‐loops (Figure 1A).^[5]^ The ROQ domain has a known affinity for stem‐loops with a trinucleotide loop, so‐called CDEs (constitutive decay elements) or a hexanucleotide loop, the ADEs (alternative decay elements).[^6^, ^7^] The currently known consensus sequences of the loops are 5’‐YRU‐3’ for CDEs and 5’‐GUUYUA‐3’ for ADEs. The composition of the stem has only minor influence on RNA recognition with a clear preference for stem lengths between 6 to 8 bp and a slight preference for purine‐stacks at the 3’‐side of the stem.^[7]^ By recognizing such stem‐loop structures in the 3’‐UTR of mRNAs, Roquin is able to reduce the half‐life of its target mRNAs. Degradation is initiated by recruitment of the Ccr4‐Not complex and subsequent deadenylation^[8]^ or recruitment of decapping factors.^[9]^ Further, Roquin proteins harbor a CCCH‐type zinc finger (ZnF) that recognizes single‐stranded AU‐rich motifs.^[10]^ ZnF binding to RNA seems to be of subordinate affinity compared to the ROQ domain, however it seems to support RNA recognition and mRNA regulation within the full‐length protein.

Roquin proteins are highly present in the immune system, where they restrict inflammation by destabilizing mRNAs such as *TNF* and *IL‐6*.^[11]^ Further, they prevent autoimmune reactions by controlling T cell maturation.^[12]^ Roquin proteins also orchestrate the differentiation of T follicular helper cells (T_FH_‐cells) by modulating the levels of the *ICOS* and *Ox40* mRNAs.^[13]^ In addition to their critical role in the immune system, Roquin proteins are ubiquitously expressed and target a multitude of mRNAs in different cell types,[^7^, ^14^] suggesting a housekeeping function for Roquin‐mediated decay.

RNA Bind‐n‐Seq (RBNS)^[15]^ is a powerful method for studying RNA‐protein interactions, allowing the identification of specific RNA sequences that interact with a given protein. It combines a binding assay with high‐throughput sequencing to provide a comprehensive map of RNA‐protein interactions. In the original RBNS workflow, a short, random RNA pool is incubated with proteins and unbound RNA is removed by a washing step. Subsequently, the bound RNA is eluted from the RNA‐protein complexes and analyzed using high‐throughput sequencing in combination with a bioinformatic pipeline for motif discovery. Compared to the iterative binding amplification of the SELEX method, which only identifies high affinity motifs, RBNS can measure high to low affinity motifs within one enrichment step.^[16]^ Importantly, a good correlation between in vitro and *in cellulo* binding has been reported.^[15]^ Compared to UV crosslinking based in cell methods such as cross‐linking and immunoprecipitation (CLIP), RBNS provides a more direct characterization of binding specificity and has been shown to be able to distinguish between true and false positive sites retrieved by in vivo crosslinking experiments.^[15]^ So far, RBNS has only been performed with short random RNA pools, but in principle different input pools should be usable,^[16]^ e.g. for the detailed study of RNA structure binding RPBs.

In this study, we introduce structured RBNS (sRBNS) that utilizes randomized RNA structures as an RNA input pool, which is the main difference compared to conventional RBNS. We developed this method to comprehensively characterize the binding capacity of the unique ROQ domain of Roquin at nucleotide resolution. This evolution of the RBNS approach is essential for investigating RNA‐protein interactions involving RNA structures. Previous CLIP^[17]^ and RBNS^[18]^ experiments were unable to retrieve the RNA‐binding properties of Roquin, specifically its preference towards different stem‐loops. Limiting factors are most likely a bias for cross‐linking to single‐stranded regions in CLIP and an underrepresentation of larger RNA structures within the fully randomized input pool in RBNS. We find strong discrimination against hairpins capped with tetra‐ and pentanucleotide loops and characterize novel CDE and ADE loop motifs that function as Roquin binding sites in vitro and *cis*‐regulatory elements in cells. Using the extended ADE loop, consensus we identify numerous new Roquin target mRNAs. Thus, we comprehensively decipher the rules of endogenous target recognition by the ROQ domain and add to the understanding of the cellular function of Roquin in the immune system and beyond. Furthermore, this study lays the basis for the use of RBNS in the in‐depth characterization of the RNA‐binding preference towards RNA structures of the multitude of yet uncharacterized RBPs.

## Results and Discussion

### sRBNS Faithfully Recapitulates the Binding Preference of Roquin towards CDEs

We used sRBNS to decipher the exact binding preferences of the coreROQ domain of Roquin‐1 towards stem‐loops with different loop sizes and sequences (Figure 1B). To test and extend the target space of accepted Roquin binding sites, we designed different RNA pools containing a mixture of structured stem‐loop elements. As so far RBNS has only been used with short, random sequence libraries without specific structural scaffolds,[^15^, ^19^] we first evaluated whether we could adapt the protocol for use with pre‐structured libraries. For that, we designed a pilot experiment to verify that sRBNS faithfully recapitulates the RNA‐binding preference of Roquin for CDEs in cells. Thus, we created an RNA pool containing two different functional CDEs from the 3’‐UTRs of *UCP3* and *TNF* as scaffolds and randomized the trinucleotide loop (Figure 1C). This yielded two CDE variants with 64 trinucleotide loop combinations each. We used two different CDEs as scaffold to buffer for artifacts by potential misfolding of individual loop variants. As protein bait, we used the coreROQ domain N‐terminally tagged with a Twin‐Strep‐tag for separation with Strep‐Tactin®‐coated magnetic beads and specific elution with the competitor biotin. We used one protein concentration, 50 nM of the coreROQ domain, which corresponds to the known K_D_s for the wild type CDE elements.^[20]^ Enriched trinucleotide loop sequences were analyzed with a custom bioinformatics pipeline.

In a first step, the occurrence of the conserved stems and the different variants of the loops is counted for each of the conditions. An enrichment factor, defined as the sRBNS E‐value, is then calculated for each occurring loop variant. Any E‐value with a z‐score >3 was considered as significantly enriched. A comparison of the conserved stem region of the pulldown library with the input shows that the *TNF* stem is ~1.4‐fold enriched against the *UCP3* stem (Figure 1D), which is consistent with its ~4‐fold higher affinity.^[20]^ Analyzing the bound loop sequences, we found a 20‐ to 28‐fold enrichment of the known wild type trinucleotide loop sequences, which are 5’‐YAU‐3’ for the *UCP3* CDE and 5’‐YGU‐3’ for the *TNF* CDE, in human and mouse (Figure 1E and F and Supporting Information Table 1). Importantly, only the four possible combinations of the known 5’‐YRU‐3’ CDE motif were significantly enriched with a z‐score >3. Thus, the sRBNS pipeline faithfully recapitulates the known preferences of the coreROQ domain towards CDEs with respect to the trinucleotide loop sequence. Further, it recapitulates the preferred directionality of the loop closing base pair, which is Y−R in the *TNF* CDE with a G in the middle position of the trinucleotide loop and R−Y in the *UCP3* CDE with an A in the middle position.^[7]^ Thus, sRBNS is superior to the conventional RBNS approach in reproducing known Roquin binding preferences, which could not achieve high enrichment values and delineate detailed binding preferences towards stem‐loops.^[18]^ Thus, in a next step, we used sRBNS to explore the full binding capacity of Roquin towards different loop motifs and sizes.

### Roquin Has a Clear Preference for Trinucleotide Loops over Tetra‐ and Pentanucleotide Loops

**Figure 2:**
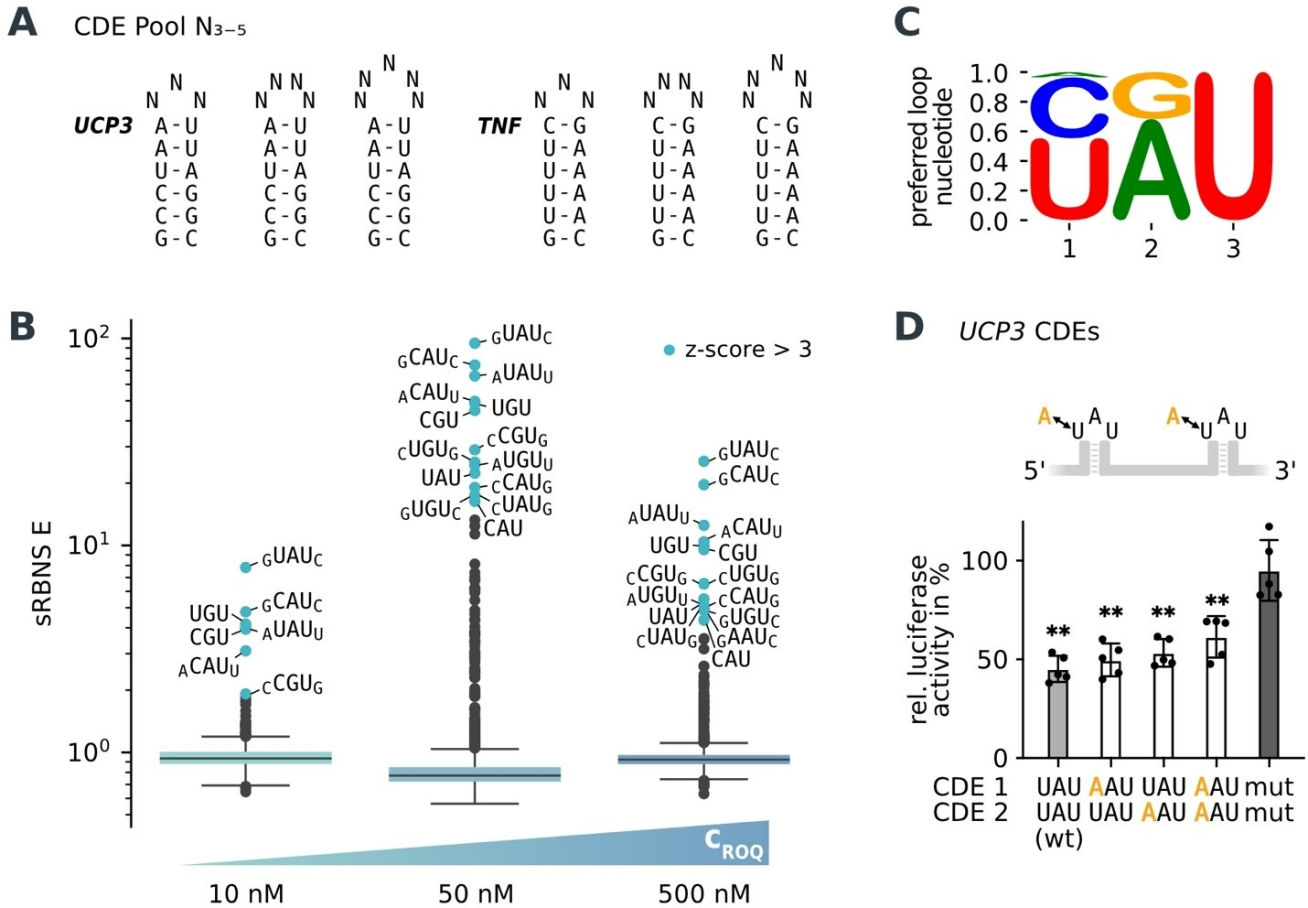
Characterization of the loop size and nucleotide composition in CDE loops. A) Input pool with different random loop sizes (N_3‐5_) on top of the CDE stems from the *UCP3* and *TNF* 3’‐UTRs. B) Boxplots of the enriched loop‐motifs against the input library (shown in A) at 10, 50 and 500 nM ROQ domain concentration. Pool size *n*=1344. Values greater than three standard deviations above the mean are highlighted in blue (z‐score >3). Lowercase letters indicate bases that form Watson–Crick base pairs. C) Preferred nucleotide logo composed of all possible trinucleotide loops weighted by their enrichment at 500 nM coreROQ domain. D) Luciferase reporter assay in HEK293 cells with wild type and mutated loops of the tandem CDE motif of the *UCP3* 3’‐UTR. Firefly luciferase activity was normalized to *Renilla* luciferase as an internal transfection control. Values are normalized to an empty vector control. *n*=5. Data are presented as mean ± standard deviation. Statistical significance was calculated by Student's t‐test (two‐tailed, paired), (**) P‐value <0.01.

Several studies suggested that Roquin proteins might also recognize stem‐loops capped with tetra‐ and pentanucleotide loops.[^14^, ^21^, ^22^] However, no high affinity interaction of Roquin with such stem‐loops has been shown. To clarify the binding competence of Roquin towards all possible tetra‐ and pentanucleotide loops, we expanded our CDE pool to include these larger loop sizes. For that, we designed an RNA pool which contains the CDEs of the 3’‐UTRs of *UCP3* and *TNF* topped with all possible sequences of three, four and five nucleotides in length (Figure 2A). To achieve the same ratios for all of the 1,344 possible loop variants, the different RNA pools were mixed with the respective excess of the more complex larger loops. In principle, the pentanucleotide loop pool already contains all variations of trinucleotide loops when base pairing between the outer loop bases occurs. However, this creates stem‐loops with by one base pair longer stems. As previous work showed that the stem length affects Roquin recognition,[^7^, ^8^] we opted for individual pools for tri‐ and pentanucleotide loops to exclude such bias.

RBNS also allows the use of several defined protein concentrations to distinguish between high and low affinity targets. Thus, to dissect high and low affinity motifs within one experiment, we used three different protein concentrations of 10, 50 and 500 nM coreROQ domain. These concentrations were chosen to account for high affinity motifs (10 and 50 nM), as reflected by the wild type CDEs and low affinity motifs (500 nM), which functional importance in cells would need to be tested. At all three concentrations, we found that the binding of trinucleotide loops with the known 5’‐YRU‐3’ motif is clearly favored (Figure 2B and Supporting Information Table 2). No tetranucleotide loop sequences were significantly enriched. Pentanucleotide loop motifs were only enriched when their outer bases can form Watson–Crick base pairs, resulting in a CDE with the known trinucleotide loop motif and a stem extended by one base pair. Pentanucleotide loops also recapitulate the preferred closing base pair rule. For example, 5’‐CUGUG‐3’ has a higher E‐value of 25.2 compared to 5’‐GUGUC‐3’ with 17.3 at 50 nM coreROQ domain. This is consistent with a middle G favoring closing base pairs with a Y−R directionality. In addition, the distribution of the individual stems shows a small enrichment towards the *TNF* CDE as in the pilot sRBNS experiment (Figure S1A). In sum, the sRBNS experiment shows a clear preference of Roquin for trinucleotide loops and faithful discrimination against all tetra‐ and pentanucleotide loops in this competition experiment.

### AAU – A Novel CDE Trinucleotide Loop Motif

At the highest protein concentration, we identified 5’‐AAU‐3’ as a novel possible CDE loop motif, which differs from the known consensus by having an A at the first position. A corresponding motif logo composed of all possible trinucleotide loops weighted by their enrichment at 500 nM coreROQ domain is shown in Figure 2C. This novel binding motif is only observed at higher protein concentrations (sRBNS E=4.9 for 5’‐GAAUC‐3’ at 500 nM coreROQ) and is less enriched compared to the corresponding wild type sequence (sRBNS E=25.5 for 5’‐GUAUC‐3’ at 500 nM coreROQ), suggesting a reduced affinity. To test if this motif is active in regulating mRNA decay in cells, we performed dual luciferase reporter assays. For that, the tandem CDE motif from the 3’‐UTR of *UCP3* ^[7]^ was mutated to include A instead of U at the first position in the trinucleotide loop of either one or both CDEs. Comparing the luciferase activity of the AAU‐CDE variants with mutations that abrogate CDE function, showed that they are *bona fide* regulatory active motifs (Figure 2D). In line with their enrichment only at higher protein concentrations, they are slightly less efficient in reduction of luciferase activity compared to the wild type 5’‐UAU‐3’ variant. Together, these experiments confirm that sRBNS is not only able to inform on RNA structures which are not recognized, but also is suitable to identify new motifs that are active regulatory elements in cells.

### The ROQ Domain Binds to Previously Unrecognized ADE Hexanucleotide Loop Motifs

**Figure 3:**
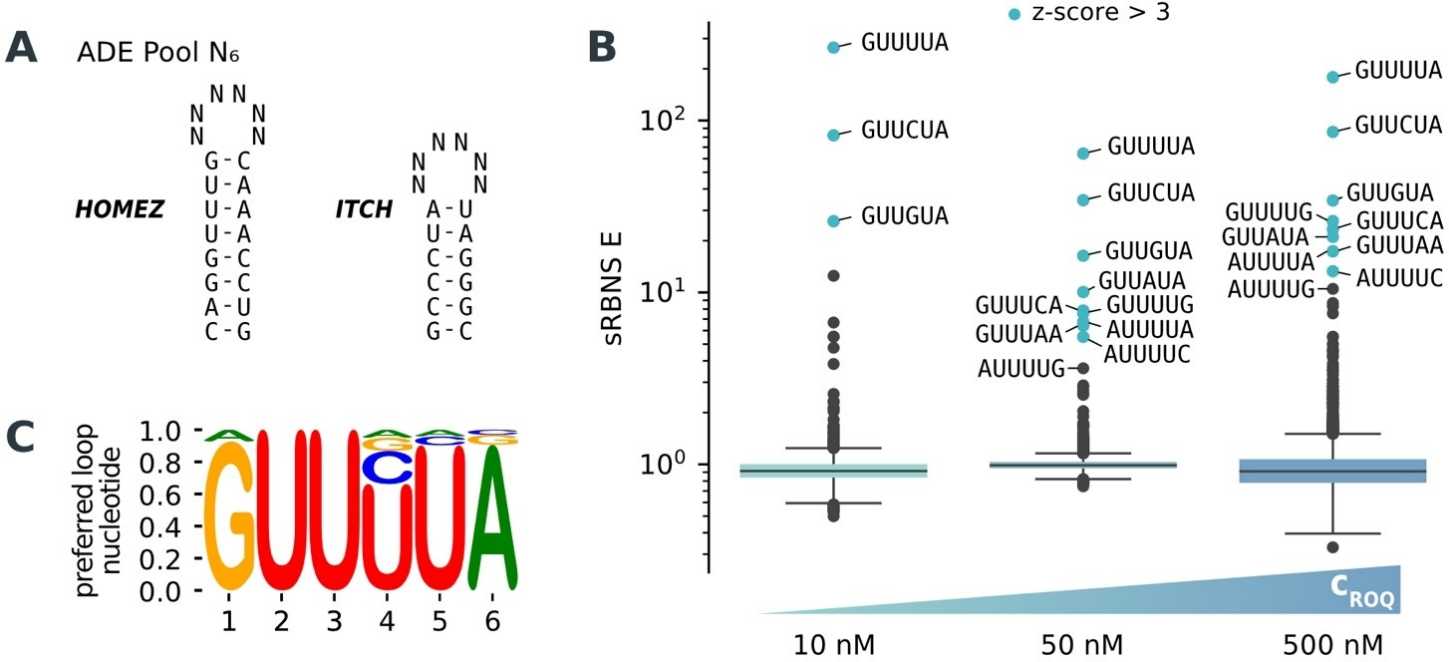
Characterization of the nucleotide composition in ADE loops. A) Input pool with different random loops N_6_ on top of the ADE stem from the *HOMEZ* and *ITCH* 3’‐UTRs. B) Boxplots of the enriched loop‐motifs against the input library (shown in A) at 10, 50 and 500 nM ROQ domain concentration. Values greater than three standard deviations above the mean are highlighted in blue (z‐score >3). Pool size *n*=4096. C) Preferred nucleotide logo composed of all possible hexanucleotide loops weighted by their enrichment at 500 nM coreROQ domain.

Based on the successful recovery of the wild type loop sequences and expansion of CDE motifs, we asked if the known ADE hexanucleotide loop motif 5’‐GUUYUA‐3’^[6]^ might also be more complex than previously recognized, with more sequences supporting Roquin recognition and ultimately regulation in cells. Thus, we designed a pool of two ADE stems each capped by all 4096 possible sequence motifs. As scaffolds, ADEs from the *HOMEZ* and *ITCH* 3’‐UTRs were used (Figure 3A).^[7]^ We decided to perform a separate experiment for ADEs, because we wanted to cover potential low‐affinity interactions that might be lost with larger input pools. We used the same protein concentrations as for the CDE library to assess high and low affinity motifs simultaneously. As wild type ADEs are bound with similar or even higher affinity than CDEs,^[6]^ we expected high affinity motifs to be enriched at 10 nM and lower affinity motifs at 50 and 500 nM of coreROQ domain. Analyzing the sRBNS experiment, we indeed identified the two wild type loop sequences (5’‐GUUUUA‐3’ and 5’‐GUUCUA‐3’ for the human *HOMEZ* and *ITCH* ADEs, respectively) to be significantly enriched at 10 nM protein concentration. Further, a third motif 5’‐GUUGUA‐3’ was significantly enriched, suggesting that there are indeed other hexanucleotide loop sequences, that are bound by Roquin with high affinity. Interestingly, there is evidence of conservation for this ADE loop variant across different species. Thus, an ADE‐like stem‐loop topped with 5’‐GUUGUA‐3’ was recently shown to mediate degradation of the *ets‐4* mRNA in *C. elegans* by the Roquin ortholog RLE‐1.^[23]^

Analyzing the higher protein concentrations, several loop sequences showed enrichment, considerably expanding the known ADE consensus (Figure 3C). Most versatile is the nucleotide at position 4, which allows for all four nucleotides with a preference of U>C≫G>A. Position 5 allows for three nucleotides with U≫C>A. Further, the outer nucleotides of the loop (positions 1 and 6) are variable allowing for all possible combinations of purines (G/A≫G/G>A/A≫A/G) as well as A/C as the least significantly enriched variant.^[17]^ In contrast, the uracils at positions 2 and 3 are completely invariant (Figure 3B and C). Of note, the minimal Roquin response element in the *NFKBID* 3’‐UTR contains six active stem‐loops, including four CDEs and one ADE‐like stem‐loop harboring a 5’‐AUUUUC‐3’ hexanucleotide loop. Thus, even the lower enriched ADE loop motifs might be regulatory active in cells, at least in conjunction with a high affinity CDE. Analyzing the stems, there is no enrichment of either the *ITCH* or *HOMEZ* scaffold when comparing the input library with the pulldown libraries (Figure S1B), suggesting an equal affinity. Further, all of the new motifs are enriched with both stem variants (Supporting Information Table 3), suggesting that the new loop sequences are active independent of stem identity. Thus, the sRBNS assay expands the ADE sequence space and reveals a new RNA binding capacity of Roquin proteins.

### New ADE Variants Are Bound with Nanomolar Affinity

We used ITC measurements to investigate the binding affinity of the coreROQ domain towards these new ADE variants and for the independent verification of the sRBNS assay. For that, we created loop mutants of the *HOMEZ* ADE. Three RNA constructs were transcribed where the variable positions 1, 4 and 5 were changed against adenine to assess the impact of these individual positions on Roquin recognition. In addition, we analyzed one construct where positions 1 and 6 are changed to A/C to assess the impact of position 6, and because this motif is the least significantly enriched motif. For all RNA constructs used in ITC the expected ADE stem‐loop fold was confirmed by NMR spectroscopy (Figure S2A and B). A comparison of imino proton fingerprint spectra showed only minor chemical shift perturbations within the two loop closing base pairs upon introducing mutations within the loop. Thus, the integrity and comparability of the global ADE fold is maintained across mutants (Figure S2B).

All tested ADE loop variants had *K*_D_ values in the low nM range and according to their enrichment in the pulldown assay, verifying the validity of the sRBNS results (Table 1 and Figure S3). The wild type loop showed the highest affinity with a *K*_D_ value of 6.0 nM. Single mutations at positions 4, 1 and 5 of the loop to an A resulted in 2.6‐, 4.9‐ and 6.6‐fold decreased affinities, respectively, compared to the wild type. Introducing a second mutation at position 6, resulted in a further 3.8‐fold decrease in affinity compared to the single mutation of position 1.

**Table 1:** Binding affinity of the new ADE motifs towards the ROQ domain. ITC results of ROQ domain titrated with point mutants in the loop of the *HOMEZ* ADE.

Loop Sequence	sRBNS E^[a]^	ITC *K*_D_ [nM] ^[b]^
GUUUUA	64.3	6.0±1.0
AUUUUA	6.7	28.9±1.5
GUUAUA	10.1	15.7±4.6
GUUUAA	6.4	39.1±11.0
AUUUUC	5.5	111.1±28.2

[a] Enrichment values are given at 50 nM coreROQ. [b] *K*_D_ values are given as mean ± standard deviation from 3–4 independent experiments.

**Figure 4:**
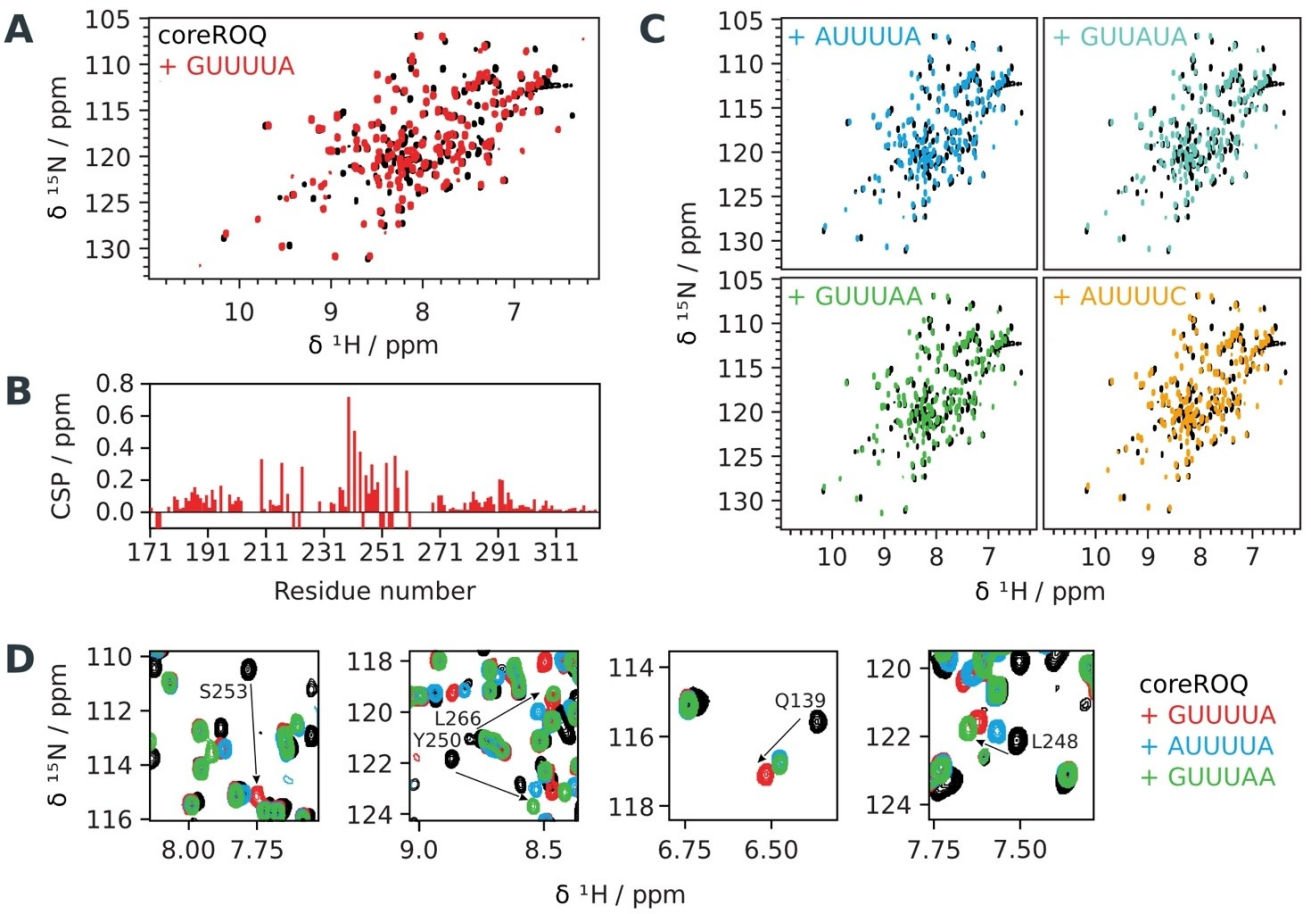
Roquin interacts with *HOMEZ* loop variants in an ADE‐like fashion. A) Overlay of ^1^H,^15^N‐HSQCs of apo coreROQ (black) in complex with wild type *HOMEZ* (5’‐GUUUUA‐3’) (red). B) CSP plot showing differences of *HOMEZ* wild type ADE with coreROQ compared to apo coreROQ (derived from overlay in panel A). C) Overlays of ^1^H,^15^N‐HSQCs of apo coreROQ (black) in complex with the different ADE variants (colored). D) Zoom‐ins of ^1^H,^15^N‐HSQC spectra shown in A and B) for 5’‐GUUUUA‐3’, 5’‐AUUUUA‐3’ and 5’‐GUUUAA‐3’ loop ADEs in complex with coreROQ. Trajectories of CSPs are indicated by arrows.

Overall, the double mutant 5’‐AUUUUC‐3’ showed the least affinity with a *K*_D_ value of 111.1 nM and mutation of position 5 had the highest impact on affinity for any individual nucleotide change. Interestingly, mutations of position 4 and 5 showed a favourable entropical contribution to binding, while RNA‐protein interaction in the wild type and mutants at position 1 and 6 are driven by strong enthalpic contributions (Figure S3), suggesting an impact on the exact binding path for these two positions. Next, we performed NMR experiments to analyze the binding mode of the coreROQ domain to the individual ADE variants at single amino acid resolution (Figure 4). The ^1^H,^15^N‐HSQC fingerprint spectrum of coreROQ in complex with the wild type *HOMEZ* ADE indicated binding in an ADE‐like^[6]^ fashion (Figure 4A), which is confirmed by a quantitative analysis of chemical shift perturbations (CSPs) (Figure 4B). In our NMR titration experiments, we observed binding for all loop variants (Figure 4C), and overall similar CSP patterns, confirming a conserved ADE binding mode. Binding of the 5’‐GUUAUA‐3’ mutant at position 4 led to an identical spectrum of the coreROQ domain compared to the wild type RNA (Figure S2C), suggesting position 4 to be less conserved. This is in line with position 4 being the most variable position in sRBNS and showing the least impact on the binding affinity (Table 1). Interestingly, the NMR spectra of complexes with the single‐mutant 5’‐AUUUUA‐3’ and the double‐mutant 5’‐AUUUUC‐3’ completely superimpose (Figure S2C). This suggests that position 1 is dominant with respect to the binding mode. Although this is not reflected in the lowest of all observed affinities of the double mutant, it is consistent with mutation of position 6 showing the second lowest individual impact on affinity. For 5’‐AUUUUA‐3’ and 5’‐GUUUAA‐3’, which had the highest individual impact on affinity, we observed significant deviations in CSPs compared to the wild type (Figure 4D). While some residues (e.g. S253) show different line broadening, other CSPs differ in size (L266, Q139, Y250, L248) or trajectory (Y250, L248) from the wild type. While for 5’‐AUUUUA‐3’ the observed CSPs are lower compared to the wild type, for 5’‐GUUUAA‐3’ some residues show similar CSPs (L266, L248), but a change in trajectory (L248, Y250). Altering position 5 had the highest individual impact on affinity but, like position 4, seems entropically favoured, suggesting a slightly different path of complex formation. Interestingly, RNA‐protein structure prediction using Alphafold3^[24]^ suggested that an A at position 5 in the loop might clash with Y250 (Figure S4), which could explain the observed change in CSP trajectory. Overall, however, alterations in the CSP size and trajectories suggest only minor deviations from the conserved ADE binding mode and are consistent with the observed high affinity recognition in the low nM range.

### ADE Variants Facilitate the Identification of Novel mRNA Targets

**Figure 5:**
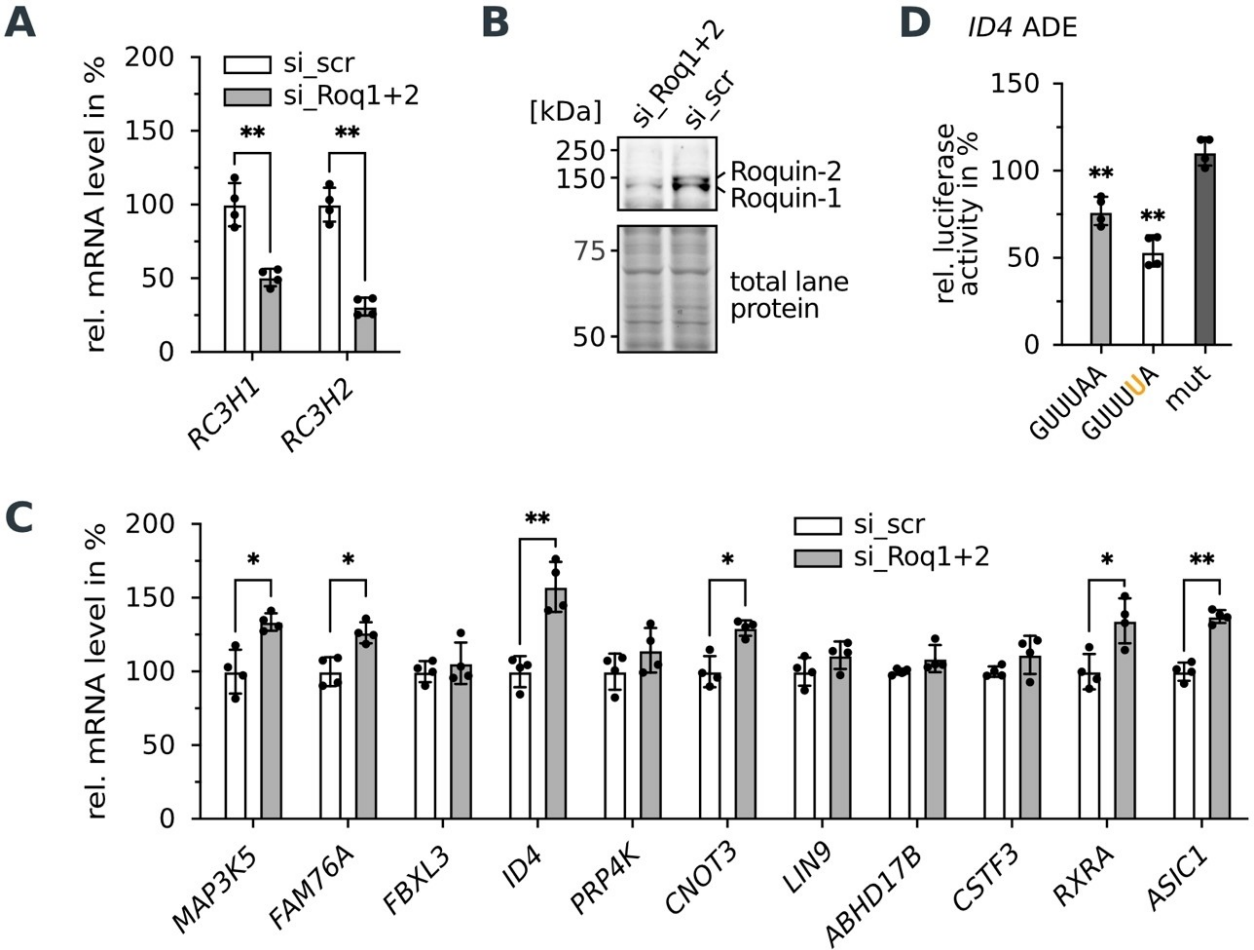
Confirmation of predicted new Roquin target mRNAs in cells. A and B) Verification of siRNA‐mediated Roquin knockdown in HEK293 cells. A) RT‐qPCR quantification of mRNA levels of *RC3H1* and *RC3H2*. Values are normalized to the housekeeping gene *RPLP0. n*=4. B) Western blot of Roquin‐1 and Roquin‐2. *n*=4. C) RT‐qPCR quantification of mRNA levels with new ADE elements after siRNA‐mediated knockdown of Roquin‐1 and Roquin‐2 in HEK293 cells. Values are normalized to the housekeeping gene *RPLP0. n*=4. D) Luciferase reporter assay in HEK293 cells with wild type and mutated loops of the *ID4* ADE variant. Firefly luciferase activity was normalized to *Renilla* luciferase as an internal transfection control. Values are normalized to an empty vector control. *n*=4. Data in A), C) and D) are presented as mean ± standard deviation. Statistical significance was calculated by Student's t‐test (two‐tailed, paired), (**) P‐value <0.01. (*) P‐value <0.05.

We identified new mRNA targets of Roquin in a bioinformatic prediction based on the hexanucleotide loop variants found with sRBNS. For that, stem‐loops with a stem length of at least six base pairs topped with the enriched hexanucleotide loop motifs derived by sRBNS (5’‐GUURUA‐3’, 5’‐GUUYMA‐3’, 5’‐GUUYUG‐3’, 5’‐AUUYUA‐3’, 5’‐AUUYUC‐3’, 5’‐AUUYUG‐3’) were predicted in all human 3’‐UTRs. This search resulted in 158 new ADE elements in 155 different candidate genes containing 1–2 ADE elements in their 3’‐UTR (Supporting Information Table 4). If these ADE variants are functional Roquin binding sites, we expect that they mediate mRNA destabilization in cells. To test whether the bioinformatically predicted binding sites are functional in gene repression, a selection of the genes, which are predicted to encode ADE variants in the 3’‐UTR of their mRNA, were tested for enhanced mRNA levels after Roquin knockdown. Candidates were selected to have a folding probability of at least 19 %, to contain different ADE motif variants and to be expressed in HEK293 cells, to facilitate mRNA quantification. We excluded, ADEs with low folding probabilities (<5%), as they may not be targeted by Roquin.^[7]^ Further, we excluded mRNAs, which also harbor a known CDE motif to exclude gene regulation by CDEs. Successful knockdown of Roquin‐1 and −2 was quantified at mRNA and protein level (Figure 5A and B, Figure S5). Five out of the tested ten candidate target mRNAs showed enhanced mRNA levels after Roquin knockdown to a similar extent as to the positive control *MAP3 K5* mRNA encoding a wild type ADE motif^[7]^ (Figure 5C). Thus, mRNAs harboring the newly identified ADE loop variations are *bona fide* targets of Roquin proteins. Importantly, mRNAs harboring ADE variants with A/C or A/G at positions 1 and 6, which were lower enriched motifs and had the lowest *K*_D_ of all tested variants, also showed regulation in response to Roquin knockdown. Previous experiments in HEK293 cells showed that not all mRNAs harboring a high affinity motif in their 3’‐UTR are induced upon Roquin knockdown, while they can be responsive in other cell types.^[7]^ One reason, might be cell type‐specific levels of RBPs, which might impact Roquin‐mediated mRNA decay, such as the co‐repressor Regnase‐1^[25]^ or competitor AUF1.^[20]^ Thus, the non‐responsive mRNAs might still be true Roquin targets depending on the cellular environment.

To verify that the observed regulation is mediated by the new ADE motifs, we tested the function of the ADE variant from the *ID4* 3’‐UTR in a dual luciferase reporter assay. *ID4*, is the fourth member of the “Inhibitors of DNA‐Binding proteins” (ID) family consisting of transcription‐inhibiting proteins.^[26]^ The *ID1‐3* mRNAs have previously been identified as Roquin targets and harbor CDE elements in their 3’‐UTRs.^[8]^ *ID4* was so far not recognized as Roquin target gene. The *ID4* ADE contains an A at position 5. The ADE along with 40 nt endogenous flanking sequence context was inserted into the 3’‐UTR of firefly luciferase. Comparison with the empty vector control shows reduction of luciferase activity by the ADE variant to ~ 75 % (Figure 5D). Destruction of the ADE structure by mutation of the stem fully restores luciferase activity. Further, capping the *ID4* ADE with the wild type loop sequence 5’‐GUUUUA‐3’ shows a stronger reduction in luciferase activity to ~ 50 %. This is in line with the stronger binding affinity of Roquin towards the wild type loop. Thus, the identified ADE motif variations are indeed functional regulatory elements that mediate mRNA decay by Roquin proteins independent of other high affinity motifs.

Taken together, we successfully identified new ADE motifs, which are bound with low nM affinity by Roquin proteins in vitro and confer gene repression in cells. Importantly, these new motifs can be used to identify new Roquin target genes and thus expand the known target space.

## Conclusion

The RNA‐binding preferences of many RBPs are still not known, precluding the detailed characterization of their cellular function as well as specific targeting by modern RNA therapeutics.^[27]^ Especially the preference towards (or discrimination against) RNA structures of RBPs is less understood compared to purely linear sequence motifs. This might be of particular interest when analyzing the RNA‐binding capacity of viral proteins or moonlighting RBPs such as metabolic enzymes, signaling receptors and transcription factors, which can specifically recognize structured RNA.[^28^, ^29^, ^30^]

Here, we introduced sRBNS, an RNA structure‐centric approach to characterize the RNA binding preference of the RBP Roquin‐1. For the first time, we showed the power of sRBNS for the characterization of protein interactions with structural RNA elements at nucleotide resolution. sRBNS accurately recapitulates the known RNA binding preference of Roquin towards CDEs, which could not be achieved with CLIP or conventional RBNS experiments. Competition experiments demonstrate a clear discrimination against tetra‐ and pentanucleotide loops and identified a new lower affinity CDE motif – 5’‐AAU‐3’ – which is active in mRNA degradation. Further, sRBNS allowed us to considerably expand the known ADE consensus. This revealed the existence of previously unrecognized ADE hexanucleotide loop motifs with the most versatile nucleotide variations at positions 4 and 5. The new loop variants bind with low nanomolar affinity to the ROQ domain and according to their enrichment factors in the sRBNS, demonstrating faithful recapitulation between low and high affinity motifs. The new motifs allowed the identification of new target genes of Roquin in cells.

Thus, sRBNS for the first time enables the detailed characterization of the RNA interactions of RBPs beyond short, single‐stranded sequence motifs and lays the basis for investigating more complex RNA‐protein‐interactions. In order to identify possible RNA structure‐recognizing proteins, the use of a mixture of pre‐structured RNA pools or the combination of structured with neighboring random regions will be viable strategies to uncover complex binding events.

## Supporting Information

The authors have cited additional references within the Supporting Information.[^31^, ^40^]

## Funding

This work was supported by the Deutsche Forschungsgemeinschaft (SFB902/B14 and WE 5819/3–1 to J.E.W.) and the U.S. National Institutes of Health (grant R35 GM145283 to D.H.M). A.S. acknowledges support through DFG grant numbers SFB902/B16, SCHL2062/2–1 and 2–2, and by the Johanna Quandt Young Academy at Goethe (stipend number 2019/AS01). The Frankfurt BMRZ acknowledges support from the state of Hesse and funding via the IWB‐EFRE‐program 20007375.

## Conflict of Interests

The authors declare no conflict of interest.

1

## Data Availability

Additional data is available as Supplemental Material. All sequencing data are available in the Gene Expression Omnibus (GEO) under the accession number GSE270841. A description and the source code of the sRBNS analysis script is available at: https://gitlab.uni‐marburg.de/fb16/ag‐weigand/srbns.
